# Tensor enhanced chest cancer classification via CNN and Vision Transformer models

**DOI:** 10.1371/journal.pone.0348863

**Published:** 2026-06-02

**Authors:** Nayab Asim, Mehreen Sirshar, Mohammad Zubair Khan, Sidra Ejaz, Sobia Khalid, Ibrahim Aljubayri, Abdulrahman Alahmadi

**Affiliations:** 1 Software Engineering, Fatima Jinnah Women University, Rawalpindi, Punjab, Pakistan; 2 Faculty of Computer and Information Systems, Islamic University of Madinah, Medina, Saudi Arabia; 3 Department of Computer Science and Information, Imam Mohammad Ibn Saud Islamic University (IMSIU), Riyadh, Saudi Arabia; 4 Department of Computer Science and Information, Applied College, Taibah University, Madinah, Saudi Arabia; 5 Energy, Industry, and Advanced Technologies Research Center, Taibah University, Madinah, Saudi Arabia; Soochow University, CHINA

## Abstract

Lung diseases, particularly lung cancer, remain a leading cause of mortality worldwide, accounting for approximately 1.8 million deaths annually. Early and accurate diagnosis is critical for improving patient outcomes. This study also introduces a unified platform for evaluating multiple convolutional neural network architectures and comparing them to a Vision Transformer model while utilizing a common tensor-based preprocessing pipeline for classifying lung cancer with CT/PET-CT imaging. To enhance model adaptability, all input images were initially converted into tensors prior to training, enabling implicit fine-tuning without altering the original architecture. The YOLOTransfer dataset, comprising diverse and annotated medical images, was used to benchmark model performance. Classical CNN models such as AlexNet, VGG-16, ResNet-50, DenseNet, and EfficientNet were compared against ViT in terms of accuracy, sensitivity, specificity, F1-score, and AUC-ROC. Among all models, ResNet-50 and EfficientNet achieved the highest accuracy, while the Vision Transformer showed competitive results in capturing complex global patterns. The findings highlight the complementary strengths of convolutional and transformer-based architectures for medical image analysis and demonstrate the feasibility of deep learning approaches for lung cancer detection.

## Introduction

The lungs are vital organs responsible for gas exchange, delivering oxygen to tissues and removing carbon dioxide, yet this essential system is highly vulnerable to malignant disease. Lung cancer remains the leading cause of cancer death worldwide, accounting for an estimated 2.5 million new cases and 1.8 million deaths in 2022 [1]. Globally, lung cancer contributes nearly one in eight new cancer diagnoses and almost one in five cancer deaths, underscoring its disproportionate impact on overall cancer burden [1]. In the United States alone, lung cancer is projected to cause roughly 350 deaths per day, remaining the foremost cause of cancer mortality despite substantial recent declines in death rates [2,3].

Lung cancer is broadly classified into non–small cell lung cancer (NSCLC), which accounts for about 80–85% of cases, and small cell lung cancer (SCLC), which is less common but more aggressive [4]. Although advances in systemic therapies and earlier stage detection have improved survival for NSCLC, overall 5-year survival for lung cancer remains markedly lower than for many other major cancers [2,5]. A key driver of this poor prognosis is that most patients are still diagnosed at advanced stages; population-based analyses show that distant-stage disease historically dominated presentations, with only about a quarter of U.S. lung cancers detected at a localized stage, even as localized incidence and survival have recently improved [3,6]. Similar patterns are observed internationally, where lung cancer continues to top cancer mortality statistics despite improvements in tobacco control and treatment [7,8].

Imaging plays a central role in early detection. Low-dose computed tomography (LDCT) screening in high-risk individuals has demonstrated substantial reductions in lung cancer mortality compared with chest radiography, leading to strong recommendations for annual LDCT screening in eligible populations [9,10]. Nevertheless, real-world uptake and adherence to screening remain suboptimal, and LDCT generates a high volume of scans with non-trivial false-positive rates and incidental findings, increasing demands on radiologists and healthcare systems [7–9]. These challenges highlight the need for robust, scalable tools to assist in image interpretation and risk stratification.

Against this backdrop, artificial intelligence (AI) particularly deep learning (DL) has emerged as a powerful approach for lung cancer diagnosis and prognosis. Convolutional neural networks (CNNs) have demonstrated high performance for detection, nodule characterization, and subtype classification from CT and other imaging modalities, often surpassing traditional machine-learning pipelines based on hand-crafted features [5,11,12]. More recent work explores transformer-based architectures, such as Vision Transformers (ViT), which leverage self-attention to capture long-range spatial dependencies, addressing limitations of CNNs’ primarily local receptive fields and enabling modeling of complex, subtle imaging patterns [11,13]. Comparative studies suggest that CNNs and ViTs offer complementary strengths, with CNNs often excelling on smaller datasets and ViT-based or hybrid models achieving state-of-the-art accuracy when sufficient data and appropriate regularization are available [11–13].

**Fig 1:**
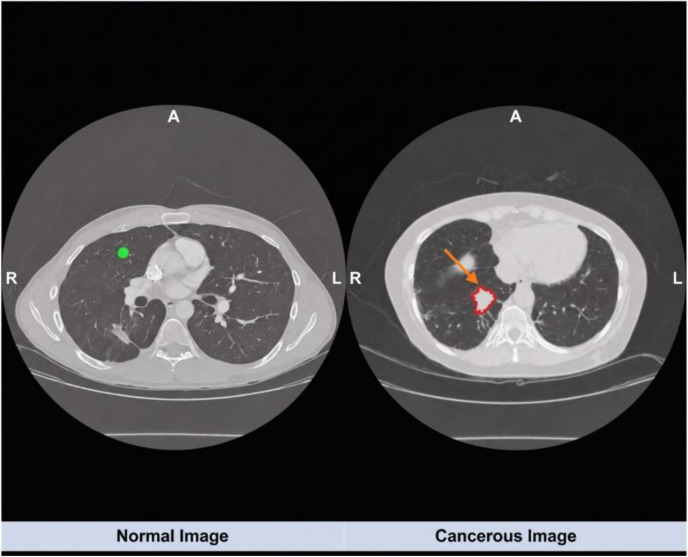
Representative lung images: (a) Normal lung scan showing clear lung fields with no visible abnormalities, and (b) Cancerous lung scan exhibiting prominent lesions and irregular opacity patterns suggestive of malignancy [6]. Images retrieved from publicly available sources via Google Images for educational and illustrative purposes.

Building on this evolving landscape, the present work systematically evaluates and compares multiple CNN architectures and a Vision Transformer model on a curated lung-cancer imaging dataset containing CT and PET-CT studies. Rather than modifying network architectures, all images are converted into tensor representations compatible with pretrained models, providing a consistent input pipeline while preserving each model’s original design. Performance is assessed using clinically relevant metrics accuracy, sensitivity, specificity, F1-score, and AUC-ROC—to provide a comprehensive view of diagnostic utility and to clarify the relative advantages of CNN and transformer-based approaches for computer aided lung cancer detection. In Fig 1 Representative lung images: (a) Normal lung scan showing clear lung fields with no visible abnormalities, and (b) Cancerous lung scan exhibiting prominent lesions and irregular opacity patterns suggestive of malignancy [14]. Images retrieved from publicly available sources via Google Images for educational and illustrative purposes.

The main contributions of this study are summarized are as follows:

- we develop a unified preprocessing framework which will convert CT and PET-CT images into tensors before training them to be compatible across multiple architectures.
- We conduct a thorough benchmarking of seven CNN architectures (AlexNet, VGG-16, ResNet-50, DenseNet, EfficientNet, GoogLeNet, NiN) in addition to the Vision Transformer model using the same dataset and preprocessing strategy.
- Empirical evaluation of each model was conducted by measuring clinically relevant performance metrics, including accuracy, recall, specificity, F1 score, and AUC-ROC.
- our study provides an analysis of how convolutional and transformer-based models for lung cancer image classification differ in their strengths, allowing healthcare professionals to determine the most appropriate learning model for lung cancer diagnostic purposes.

The remaining sections are structured as follows: Section 3 of this study outlines the proposed methodology, detailing the model architecture and approach employed for lung cancer detection. Section 4 describes the experimental setup, including the dataset specifications, training parameters, and the evaluation metrics used to assess performance. In Section 5, the results are presented and analyzed, with a particular focus on key performance indicators such as the AUC, ROC curves, and confusion matrix to evaluate and compare the effectiveness of the transformer based model.

## 1. Related work

Deep learning, particularly convolutional neural networks (CNNs), has transformed medical image analysis, achieving state-of-the-art performance in lung cancer detection using chest X-ray (CXR) and computed tomography (CT) scans. The YoloTransfer dataset [15], curated specifically for lung nodule detection and classification, has supported numerous CNN-based research efforts in this domain. Lung cancer remains one of the leading causes of cancer mortality worldwide, motivating extensive work on computer-aided diagnosis and prognosis using CT and PET/CT imaging with deep learning. Early research established the effectiveness of convolutional neural networks (CNNs) for lung cancer detection, segmentation, and classification, with augmented CNNs on LIDC-IDRI achieving around 95% accuracy for benign–malignant classification, demonstrating the potential of data augmentation to improve robustness on limited CT datasets [16]. Subsequent CNN-based CAD systems further advanced performance: ensemble CNNs on IQ-OTH/NCCD reached 98.17% binary accuracy and 95.43% three-class accuracy (normal/benign/malignant), highlighting the strength of combining multiple convolutional models to approach radiologist-level performance on small datasets [17]. Custom architectures such as Enhanced CNNs and Xception-based classifiers have reported accuracies above 99% and even 100% on multi-class CT datasets, underscoring the power of transfer learning and careful preprocessing, though often in relatively constrained experimental settings with limited external validation [18,19].

Systematic reviews confirm that deep CNNs consistently outperform traditional machine learning approaches across lung nodule detection, segmentation, and classification tasks, achieving nodule detection rates above 95% and classification accuracies up to 99% on CT, while also emphasizing challenges of dataset scarcity, annotation variability, and poor generalizability to multicenter cohorts [20–22]. To improve three-dimensional context modeling, 3D architectures such as 3D-VNet for segmentation and 3D-ResNet for classification have been proposed, achieving Dice scores above 99% on LUNA16 for nodule segmentation and over 99% classification accuracy with excellent sensitivity and specificity, thereby demonstrating that volumetric modeling can substantially enhance early nodule characterization [23]. In parallel, dual-modality 3D CNNs that jointly process PET and CT volumes have been developed for automated tumor segmentation, with dual-path architectures achieving Dice scores around 0.83 and being judged clinically acceptable by radiation oncologists for radiotherapy planning [24]. PET/CT-based CNN CAD systems have also shown strong performance in multi-class settings, with architectures such as Res-SE Net achieving over 90% accuracy for differentiating NSCLC and SCLC and maintaining high performance on external test sets, indicating robustness across scanners and institutions [25].

More recently, transformer-based models and hybrid CNN–Vision Transformer (ViT) architectures have emerged to better capture global contextual information in lung imaging. Lightweight ViT designs such as gSC-DViT, which combine groupwise separable convolutions, dual attention, and transformer blocks, have achieved accuracies above 99.5% on multiple CT datasets while keeping parameter counts relatively low, suggesting that carefully constrained ViT variants can be both effective and efficient for lung cancer classification [13]. Hybrid models integrating InceptionNeXt CNN blocks with grid and block attention–based ViTs have likewise reported accuracies of 99.54% and 98.41% on IQ-OTH/NCCD and Chest CT datasets, respectively, outperforming state-of-the-art pure CNN and ViT baselines and highlighting the benefit of combining local and global feature modeling for complex nodule patterns and subtype classification [26]. Other hybrid LEViT-based frameworks have extended this idea across imaging modalities, simultaneously handling CT and histopathology images and achieving around 99% accuracy with high Matthews correlation, while providing Grad-CAM-based visual explanations to improve interpretability for clinicians [27].

Pure transformer approaches have also been explored in PET/CT for both detection and staging. A DETR-based framework has been shown to automatically localize lung tumors and classify TNM T-stage and histologic subtype on Lung-PET-CT-Dx with high accuracy (IoU ≈ 0.8 and T-stage accuracy 0.97), outperforming prior non-transformer methods for both detection and staging tasks [28]. For prognostic modeling, transformer-based and hybrid CNN–ViT models using 18F-FDG PET/CT have been developed to predict distant metastasis in NSCLC; a ViT-based multimodal model integrating PET and CT features achieved an AUC of 0.882 in validation, significantly outperforming a ResNet-50 CNN baseline and demonstrating the value of global attention mechanisms in capturing complex metastatic patterns [29]. At the classification level, comparative studies that systematically evaluate CNNs, ViTs, and hybrid architectures on multiple public CT datasets (IQ-OTH/NCCD, SPIE-AAPM-NCI, CT-Scan) using rigorous cross-validation and statistical testing have shown that ViT-based models such as SwinV2 and hybrid ConvNeXtV2 can deliver clinically high performance and often rank among the top models, though gains depend heavily on augmentation and training regimen [30].

Concurrently, a broader methodological literature explores advanced machine learning paradigms for lung cancer imaging, including reinforcement learning, GAN-based data augmentation, meta-learning, and ensemble learning. These techniques have been reported to improve accuracy, sensitivity, and specificity, particularly in low-data or imbalanced scenarios, by enriching training distributions and improving adaptation to underrepresented cancer subtypes [31]. Complementary work emphasizes the need for explainable AI, multimodal integration (e.g., combining imaging with clinical text using transformer-based NLP models), and rigorous external validation to move from promising technical results toward deployable clinical decision support [20,22]. Taken together, the literature indicates that CNNs provide strong baselines for lung cancer detection and classification, especially on limited datasets, while transformer and hybrid CNN–ViT architectures offer complementary strengths in modeling long-range dependencies, multimodal fusion, and complex global patterns. This justifies unified experimental platforms that benchmark multiple CNN backbones (e.g., AlexNet, VGG-16, ResNet-50, DenseNet, EfficientNet) against ViT-based models using consistent preprocessing and evaluation protocols, as such platforms can systematically characterize performance trade-offs across architectures, imaging modalities, and clinical tasks relevant to lung cancer diagnosis and staging. The findings of seven previous research projects (using CT scans) of lung cancer classification and detection via deep neural networks; their strengths, weaknesses, and performance metrics are summarised in Table 1.

**Table 1:** Summary of seven recent deep learning studies for lung cancer detection and classification using CT imaging.

Paper (7 recent)	Key findings	Strengths	Weaknesses / limitations	Accuracy / AUROC (if given)
Deep Learning Innovations in the Detection of Lung Cancer: Advances, Trends, and Open Challenges (2025) [32]	2018–2023 state-of-the-art survey; CNNs dominate, transformers and hybrids emerging; highlights explainable AI and data scarcity.	Very up-to-date systematic synthesis; covers multiple datasets and DL families.	No unified experimental benchmark; relies on reported (often non-comparable) metrics.	Reports many works with Acc up to 99% and AUC ~0.97, but no single pooled value.
Deep Learning Techniques for Lung Cancer Diagnosis with CT Imaging: A Systematic Review (2015–2024) [20]	Reviews 80 CT-based DL studies; nodule detection >95% (≤4 FP/scan), classification up to 99% (Sen 98%).	PRISMA-based systematic review; clear breakdown of detection/segmentation/classification.	<15% of models validated on multicenter/diverse cohorts; generalizability limited.	Example: MVSA-CNN on LIDC-IDRI Acc 97.10, Sen 96.31, Spe 97.45.
Attention Enhanced InceptionNeXt-Based Hybrid DL Model for Lung Cancer Detection (CT) [26]	Hybrid CNN + ViT with grid/block attention; multi-class (subtypes) CT classification.	Very high performance on two public datasets; lightweight (~18.1M params).	Trained on limited public datasets; no real-world clinical validation yet.	IQ-OTH/NCCD Acc 99.54%; Chest CT Acc 98.41%.
Deep Learning Techniques for Lung Cancer Recognition (CT, VGG-16 etc.) [33]	Compares CNN, VGG-16, VGG-19 transfer learning for CT-based recognition.	Simple, interpretable benchmark; shows benefit of transfer learning.	Single dataset; moderate sample size; only CNN family evaluated.	Best (VGG-16) Acc 95%.
Benchmark of DL Approaches to Predict Lung Cancer Risk (NLST, 2D vs 3D) [34]	Evaluates 21 SOTA 2D/3D models on NLST for malignancy risk.	Real screening cohort; fair head-to-head comparison under same pipeline.	Subset of 253 patients; risk-prediction only (not full CAD).	Best 3D AUROC 0.86; best 2D AUROC 0.79.
Evaluation of Recent Lightweight DL Architectures for Lung Cancer CT Classification [35]	Tests MobileOne-S0, FastViT-S12, MambaOut-Femto on public/private CT.	Focus on efficiency (params, FLOPs, latency); compares to ResNet, Swin.	Datasets relatively small (95 and 274 cases); no external hospital validation.	MambaOut-Femto: Acc 0.896 (AUC 0.972) on D1; Acc 0.916 on D2.
FPA-Based Weighted Ensemble of CNNs for Lung Cancer CT Classification [12]	Flower-Pollination-optimized ensemble of VGG16, ResNet101V2, InceptionV3.	Ensemble clearly outperforms best single CNN; adaptive weighting strategy.	Single dataset; overfitting risk; no comparison to transformers/hybrids.	Ensemble Acc 98.2%, Prec 98.4%, Rec 98.6, F1 = 0.985; best single CNN Acc 94.6%.

**Research Gap:** while numerous studies have explored the use of convolutional neural networks (CNNs) and transformer-based models in medical image analysis, limited work has been done to directly compare their performance on lung disease classification using the same dataset and experimental conditions. Most existing research either focuses on a single model type or lacks a consistent evaluation framework across architectures. Moreover, although Vision Transformers (ViTs) have shown promise in general image classification tasks, their effectiveness in lung cancer detection remains under-investigated in comparison to established CNN models. Additionally, few studies have explored the impact of tensor-based input transformation as a form of implicit fine-tuning. This study addresses these gaps by presenting a unified framework for comparing fine-tuned CNNs and ViT on the YOLOTransfer dataset using clinically relevant evaluation metrics.

## 2. Methodology

This study employs a unified methodology to evaluate and compare the performance of nine deep learning architectures, including eight Convolutional Neural Networks (CNNs) and one Transformer-based model, for binary classification of lung cancer using CT and PET-CT scan images. The models include AlexNet, VGG-16, ResNet-18, ResNet-50, DenseNet-121, EfficientNet-B0, GoogLeNet (Inception V1), Network-in-Network (NiN), and the Vision Transformer (ViT). Each architecture was chosen for its unique design features and prior success in image classification tasks. To ensure consistent evaluation, all models were trained and tested under an identical experimental setup. The input images were preprocessed with resizing 224×224formostmodelsand227×227forAlexNetandGoogLeNet, normalization, and transformation into tensors. This tensor conversion step implicitly acts as a form of fine-tuning, aligning image inputs with the model expectations and enhancing learning without modifying the internal architecture.

All CNN models were implemented using PyTorch and pretrained weights (where applicable) were fine-tuned on the YOLOLungs dataset. The training loop included 15 epochs, a batch size of 32, and optimization via the Adam optimizer with a learning rate of 0.0001. Cross entropy loss was used as the objective function. For models like AlexNet, the final classifier layer was modified to match the number of output classes, and early convolutional layers were frozen to retain pretrained knowledge. Metrics such as accuracy, specificity, classification report, and AUC-ROC curves were calculated on the test set. AlexNet was selected for its historical significance as a foundational CNN with five convolutional and three fully connected layers. VGG-16, known for its deep architecture and small 3×3 filters, was included for its strong feature extraction capability. ResNet-50 introduced residual connections to combat vanishing gradients, with ResNet-50 offering deeper and more robust learning via bottleneck blocks. DenseNet-121 connects every layer to all subsequent layers, enhancing feature reuse and gradient flow. EfficientNet-B0 scales depth, width, and resolution efficiently. GoogLeNet employed inception modules for multi scale feature extraction, and NiN used micro MLPs in place of traditional convolution filters to increase representational power. Capsule Networks (CapsNet) preserved spatial hierarchies through dynamic routing, offering robustness to affine transformations.

For transformer-based modeling, the ViT_Base_Patch16_224 model from the TIMM library was utilized. The model was initialized without pretrained weights and trained from scratch on the YOLOLungs dataset. ViT divides each image into fixed size patches and feeds the flattened patch embeddings to a standard transformer encoder. This approach leverages self-attention to capture global dependencies within the image, making it suitable for recognizing dispersed or subtle lung anomalies. The ViT model was trained for 15 epochs using the Adam optimizer and cross entropy loss. Evaluation included test accuracy, classification report, specificity per class, overall specificity, and AUC-ROC curves. The results indicated that ViT could generalize well to medical imaging tasks when trained under consistent preprocessing and evaluation conditions.

**Fig 2:**
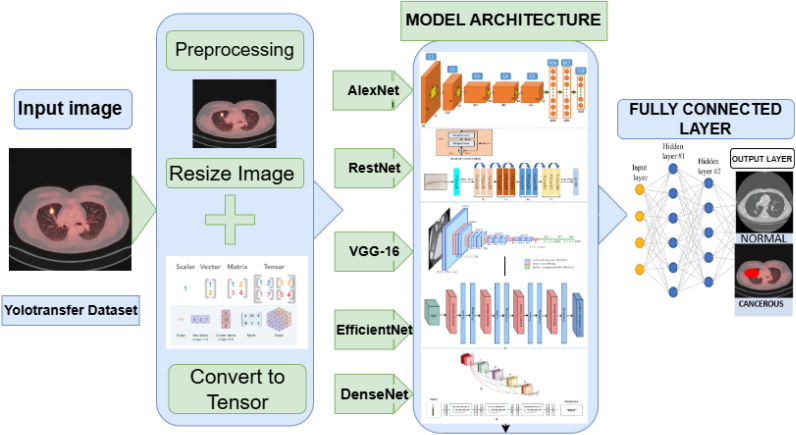
A framework pipeline for lung cancer detection using CT scan images from the YOLOTransfer dataset. The input images undergo preprocessing (resizing and tensor conversion) before being passed through five CNN models: AlexNet, ResNet, VGG-16, EfficientNet, and DenseNet. Extracted features are processed by fully connected layers to classify scans as Cancerous or Normal. Some images used in this figure were adapted from Google image search.

**Fig 3:**
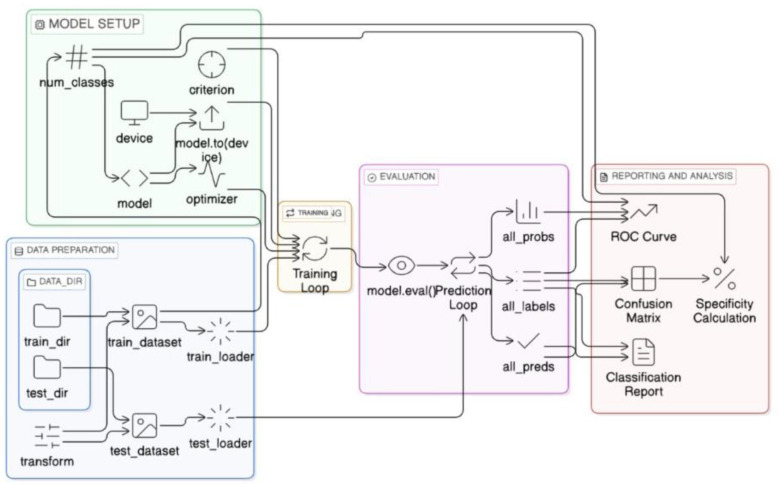
Pipeline for lung cancer classification, illustrating data preparation, model setup, training, evaluation, and performance reporting. The framework ensures consistency across CNN and transformer models, from tensor-based input transformation to final metric generation.

Figs 2 and 3 illustrates the complete model comparison pipeline, highlighting the standardized input transformation, model training, and evaluation strategy used across all architectures.

**Fig 4:**
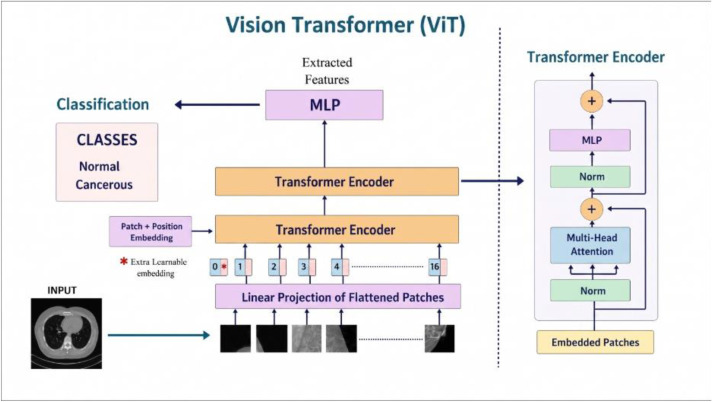
Architecture of the Vision Transformer (ViT) model used in this study. The input image is first divided into fixed-size patches, which are flattened and linearly embedded. A special [CLS] token is prepended to the sequence, and positional embeddings are added to retain spatial information. This sequence is then processed by a stack of transformer encoder layers comprising multi head self attention (MSA) and feed-forward networks (FFN). The final classification is performed using the output corresponding to the [CLS] token. This transformer-based approach enables global contextual modeling, which is especially beneficial for detecting dispersed patterns in lung imaging.

Fig 4 provides a visual representation of the Vision Transformer (ViT) architecture utilized in this study. The diagram illustrates the entire processing pipeline from the raw input image to final classification. Initially, each input image is resized and divided into fixed-size non-overlapping patches (e.g., 16×16 pixels), which are then flattened and linearly embedded into a lower-dimensional space. These patch embeddings are concatenated with a learnable class token and enriched with positional encodings to retain spatial order. The resulting sequence is passed through multiple transformer encoder blocks, each consisting of multi-head self-attention and feed-forward layers. This mechanism enables the model to capture long-range dependencies and contextual information across the entire image. The final class token output is used for classification through a fully connected layer. This architecture departs significantly from traditional CNN-based approaches by treating the image as a sequence rather than a grid, thereby facilitating global feature learning crucial for subtle pattern recognition in medical imaging.

**Algorithm 1. CNN-Based Image Classification**



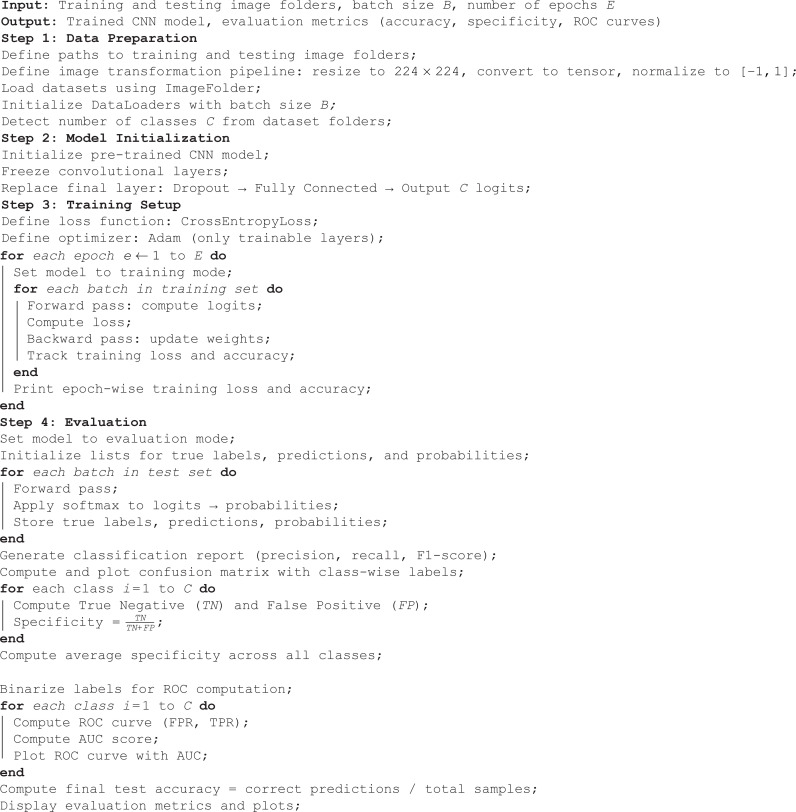



This algorithm defines a comprehensive pipeline for training and evaluating a convolutional neural network (CNN) for multi-class image classification. It begins by preprocessing the images via resizing, normalization, and batching. A pretrained CNN is initialized with frozen feature extractor layers, and its final classification layer is replaced to match the number of classes. The model is trained using cross-entropy loss and the Adam optimizer, and its performance is tracked over epochs. After training, the model is evaluated on a separate test set using multiple metrics: classification report, confusion matrix, specificity per class, overall test accuracy, and ROC-AUC scores for each class. This generic structure supports plug-and-play functionality for different CNN models and ensures reproducibility and interpretability of results in medical image analysis tasks.

**Algorithm 2. Lung Disease Classification using Vision Transformer (ViT)**



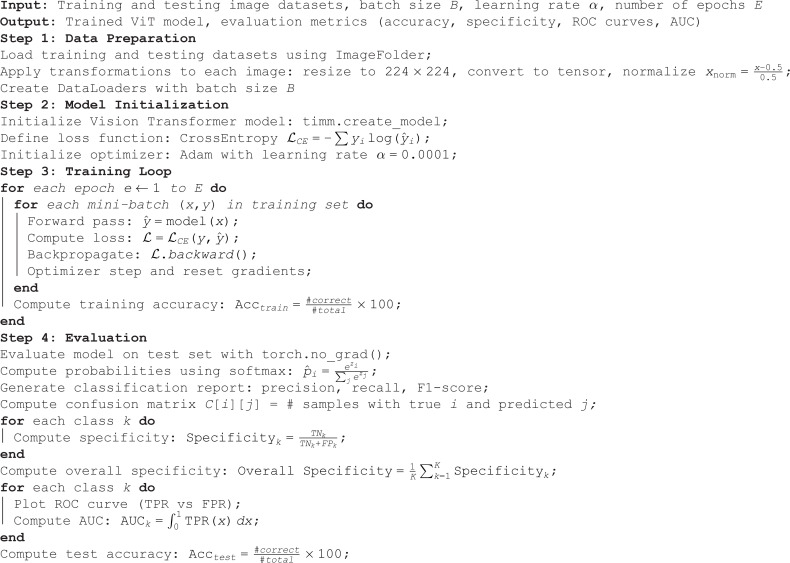



The algorithm outlines the complete workflow for lung disease classification using a Vision Transformer (ViT) model. It begins by loading and preprocessing medical image datasets, which includes resizing, normalization, and batching. The Vision Transformer is initialized using a pre-trained configuration and adapted for multi-class classification. The model is trained using the Cross-Entropy loss function and the Adam optimizer over multiple epochs, with accuracy tracked at each step. After training, the model is evaluated on a test set to compute metrics such as precision, recall, F1-score, and overall test accuracy. A confusion matrix is generated to understand the prediction distribution, and specificity is calculated for each class to assess the model’s ability to correctly identify negative cases. Finally, ROC curves and AUC scores are plotted for each class to evaluate the model’s discriminative performance across all disease categories. This step-by-step process ensures the model is not only accurate but also interpretable and reliable in detecting various lung conditions.

### 2.1 Training configuration

This study evaluated different deep learning models with the same experimental layout to provide an equable comparison between the systems, training them with 15 epochs due to limitations in CPU processing. Longer training times could yield further improvements in terms of convergence rates, but the established configuration enabled benchmarking all of the architectures evaluated consistently. The choices for learning rate, batch size, and optimizer settings were based upon previously established, commonly accepted protocols and tested in published deep learning literature. Each model utilized the same preprocessing pipeline, with all input images converted to tensors before model training. For comparative purposes between architectures, training and validation splits were identical for each model. Evaluation of the model’s performance was attempted using a common test dataset with performance characterized using multiple metric measurements, including accuracy, recall, specificity, F1 score and AUC-ROC. Future work will include extensive hyperparameter and uses of GPU-based processing to optimize performance within deeper network architectures.

### 2.2 Ethical statement

This study uses publicly available medical imaging data. No personally identifiable patient information was used, and all procedures comply with ethical standards for research involving medical data. There is no conflict of interest between authors.

## 3. Experimental setup

This section offers a comprehensive discussion of the key components involved in the experimental setup, including a detailed description of the dataset, the specific Training and implementation details.

### 3.1 Dataset

The **YoloTransfer** dataset [15], containing 5,121 publically available medical images used in lung cancer detection by Mehmet Fatih Akca using Roboflow Universe. The images are acquired from CT and PET CT DICOM scans of patients who are suspected to have lung cancer, with each image having associated XML annotation files for the bounding box of the tumor on the image. The dataset includes many of the most common histological types of lung cancers such as adenocarcinoma, small cell carcinoma, large cell carcinoma and squamous cell carcinoma. For the purposes of this study, the images in the dataset contained only two primary classes: images in the **Normal class** and in the **Cancerous class**. The Cancerous category includes aggressive lung cancer types such as adenocarcinoma and small cell carcinoma, while the normal category contains cases which are benign or non-cancerous in nature. In total, the YoloTransfer dataset consists of 5,121 images and breaks down into separate training, validation and test datasets of 3585, 1024, and 512 images respectively. This distribution provides a methodical way of evaluating the models for training and evaluation, as both training and evaluation sets are kept separate to reduce the chance of data leakage. For example, all images that belong to one patient were excluded from the other set; therefore, any model trained on this dataset will not have the ability to memorize the specific characteristics of a single patient’s image. Consequently, these models will only learn general patterns or characteristics, thus enabling them to be applied to other unknown patients. All CT and PET-CT images were formatted identically as tensors and used the same preprocessing pipeline prior to their application as inputs into the deep learning algorithms. In addition, since all images were preprocessed using a common pipeline, this allows compatibility across all architectures and models built and tested in this study. The CT images provided had a resolution of 512 x 512 pixels, with a pixel dimension of 1 mm x 1 mm; whereas the pixel resolution and dimensions for the PET images were 200 x 200 pixels, and 4.07 mm x 4.07 mm respectively. Both sets of data were collected using a variety of techniques including plain scans, contrast images and 3D reconstruction techniques, with a slice thickness of 2 mm between slices and a distance between slices between 0.625 mm and 5 mm. Patient fasting was required for at least 6 hours prior to scanning, ensuring that blood glucose levels remained at less than 11 mmol/L. Patients completed their imaging scans approximately 60 minutes after their ^18^F-FDG dose was administered (dose range: 168.72 MBq – 468.79 MBq) with an average time for 70.4 minutes. While the majority of clinical diagnoses for lung cancer are done with multi-class classification and staging of tumours, this study focuses solely on evaluating the comparative performance of deep learning architectures for binary classification (Normal vs Cancerous) in a well-controlled experimental setting. The dataset contains a large variety of imaging conditions and pathological patterns so it can be used as a tool to validate the generalisation ability of deep learning models for the detection and classification of lung cancer.

### 3.2 Training and implementation details

The training and evaluation of all models, including both convolutional neural networks (CNNs) and the Vision Transformer (ViT), were conducted using the PyTorch deep learning framework on Google Colab with CPU acceleration enabled. The dataset included chest X-ray and CT scan images, which were preprocessed according to the input requirements of each individual model. Specifically, AlexNet and GoogLeNet required input images resized to 227×227 pixels, while ResNet-50, DenseNet, VGG-16, EfficientNet, Network-in-Network (NIN), and ViT used 224×224 pixel inputs. For models pretrained on ImageNet namely ResNet-50, DenseNet, VGG-16, EfficientNet, AlexNet, GoogLeNet, and ViT image normalization was performed using a mean of [0.485, 0.456, 0.406] and a standard deviation of [0.229, 0.224, 0.225]. In contrast, NIN and CapsuleNet, which were trained from scratch, were normalized using dataset specific mean and standard deviation values computed from the training data. All models were trained using the cross-entropy loss function, which is well suited for binary classification tasks. The Adam optimizer, with a learning rate of 0.0001, was employed to update model weights. Each model was trained for 15 epochs with a batch size of 32. Data loading and shuffling during training were handled using the PyTorch DataLoader class to ensure efficient batch management and variability during training. All experiments were conducted in a single run due to computational constraints. Future work will include multiple runs and statistical averaging to improve robustness of the reported results.

Each architecture AlexNet, ResNet-50, VGG-16, DenseNet, EfficientNet, GoogLeNet, NIN, and ViT was trained on the same training split and evaluated on a consistent test set. The performance of each model was evaluated using multiple metrics: Accuracy, precision, recall, F1-score, specificity, and AUC-ROC. This unified and architecture aware experimental setup ensured a fair comparison and reliable benchmarking across all models, including both traditional CNNs and transformer based architectures like ViT.

## 4. Results

To evaluate the performance of our proposed lung cancer detection framework, we conducted extensive experiments using multiple convolutional neural network architectures, including AlexNet, VGG-16, DenseNet, ResNet, EfficientNet, GoogLeNet, Network-in-Network (NiN), and Capsule Network. These models were trained and evaluated on the YoloTransfer lung cancer dataset, which includes two classes: Normal and Cancerous. The dataset was divided into training and testing subsets, and all models were trained using identical preprocessing steps and hyperparameter configurations for fair comparison.

To comprehensively evaluate and compare model performance, we used multiple metrics: accuracy, precision, recall, F1-score, and area under the ROC curve (AUC). Furthermore, confusion matrices and ROC curves were generated for visual insight.

### 4.1 Classification performance evaluation

In this section, we present the evaluation results of the VIT transformer and various CNN architectures implemented in this study, including AlexNet, ResNet-50, ResNet, VGG-16, DenseNet, EfficientNet, GoogLeNet, NIN. The models were evaluated based on a range of performance metrics, including accuracy, recall, specificity, precision, F1-score, and AUC-ROC show in Table 2.

**Table 2:** Performance comparison of various CNN architectures and Vision Transformer (ViT) on lung cancer classification.

Model	Accuracy (%)	Recall (%)	Specificity (%)	Precision (%)	F1-score (%)	AUC-ROC (%)
AlexNet	91	88	85	96	91	97
ResNet-50	86	73	72	83	76	91
VGG-16	79	50	50	38	43	83
DenseNet	84	69	93	83	73	89
EfficientNet	85	67	66	80	70	89
GoogLeNet	50	49	48	49	48	49
Network-in-Network (NiN)	79	50	50	40	44	50
Vision Transformer (ViT)	93	81	81	83	82	89

Table 2 summarizes the performance of each model across multiple metrics. The Vision Transformer (ViT) achieved the best overall performance with an accuracy of 93%, recall of 81%, specificity of 81%, precision of 83%, F1-score of 82%, and AUC-ROC of 89%, outperforming all CNN-based models. Among CNNs, AlexNet delivered the highest accuracy (91%) and strong recall (88%) and precision (96%), resulting in an excellent F1-score (91%) and the top AUC-ROC of 97%. DenseNet followed with balanced and competitive performance across metrics, including a notably high specificity (93%). EfficientNet also showed solid results with 85% accuracy, precision of 84%, and a respectable AUC of 90%.

### 4.2 ROC curve evaluation

**Fig 5:**
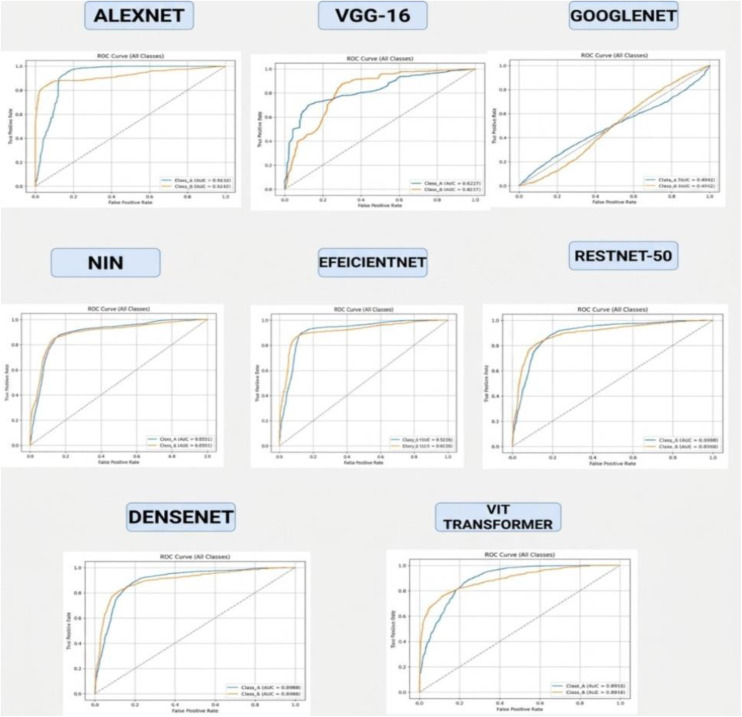
ROC curves of all CNN models (AlexNet, ResNet, ResNet-50, VGG-16, DenseNet, EfficientNet, GoogLeNet, NIN and VIT) used for lung cancer classification. The blue line shows the AUC for the cancerous class, while the orange line represents the AUC for the normal class. These curves highlight each model’s sensitivity and specificity. A higher AUC indicates better classification performance.

The Receiver Operating Characteristic (ROC) curve is a critical diagnostic tool for assessing a model’s ability to distinguish between classes. It visualizes the trade-off between the True Positive Rate (Recall) and the False Positive Rate, providing insight into model performance across various decision thresholds. The Area Under the Curve (AUC) quantifies this performance, with higher AUC values indicating superior classification capability. in Fig 5 ROC curves of all CNN models (AlexNet, ResNet, ResNet-50, VGG-16, DenseNet, EfficientNet, GoogLeNet, NIN and VIT) used for lung cancer classification. The blue line shows the AUC for the cancerous class, while the orange line represents the AUC for the normal class. These curves highlight each model’s sensitivity and specificity. A higher AUC indicates better classification performance. As shown in Fig 5, AlexNet achieved the highest AUC-ROC of 97%, closely followed by DenseNet (89%), EfficientNet (90%), and the Vision Transformer (89%). The curves for these models are steep and skewed toward the top left, indicating strong classification power. While ViT slightly underperformed AlexNet in AUC, its consistent and high performance across all metrics demonstrates its robust generalization and feature learning capabilities, especially in comparison to older CNNs.

### 4.3 Comparative insights

Comparing all models, Results shows that multiple models have different strengths that complement other models’ strengths. Specifically, vision transformers excel at capturing long-range relations while other CNN architectures (e.g., AlexNet and ResNet-50) also achieved comparable or better AUC levels) than vision transformers; thus, using both types of architectures will continue to be important for classification of medical images. However, models like GoogLeNet, and NiN exhibited performance bottlenecks, failing to capture subtle patterns essential for medical diagnosis.

## 5. Discussion

In this study, we implemented and compared a wide range of deep learning models, including classical CNN architectures and the Vision Transformer (ViT), for lung cancer detection using CT and PET-CT scan images. The CNN based models such as AlexNet, VDD-16, ResNet, DenseNet, EfficientNet, GoogLeNet, and NIN demonstrated solid performance in learning local patterns and texture features from medical images. Each CNN brought specific strengths DenseNet’s feature reuse, ResNet’s residual learning, and EfficientNet’s parameter efficiency contributing to improved classification outcomes under different computational constraints. However, CNNs are inherently limited by their localized receptive fields and struggle to model long range dependencies, which are often crucial in identifying subtle or spatially distant anomalies in medical images. To address this limitation, we incorporated a Vision Transformer (ViT), which employs self-attention mechanisms to model global contextual relationships across the entire image. Unlike traditional CNNs, ViT treats the image as a sequence of patches, allowing it to focus on both local and global features simultaneously. A key methodological enhancement in this study involved the standardized transformation of input images into tensors, serving as a form of implicit fine tuning. This transformation allowed all models, including ViT, to better align with the structural expectations of the architectures, optimizing the learning process without altering internal layers. This approach proved particularly beneficial for the transformer model, which relies heavily on structured input representations. The experimental setup maintained uniformity in data preprocessing, training loops, and evaluation metrics, ensuring a fair comparison across all models. Performance was measured using clinically relevant indicators, including accuracy, precision, recall, specificity, F1-score, and AUC-ROC. ViT demonstrated strong generalization and consistent results across evaluation metrics,Interestingly, some CNN models such as AlexNet achieved comparable or even superior performance to other models regarding certain metrics such as AUC, suggesting that different architectures can provide many different strengths rather than only one strong model especially in identifying dispersed cancerous regions, suggesting its potential for clinical applications, although further validation on external datasets is required. While transformer based models offer significant representational advantages, their training remains resource-intensive and sensitive to dataset size. Transfer learning, tensor transformation, and data augmentation played crucial roles in mitigating these limitations, improving stability, and enhancing model generalization. Nevertheless, challenges persist in scaling these models for use in resource-constrained healthcare settings. In spite of producing some useful information, this research is also limited by its dataset size and inability to extrapolate or produce results with a binary classification (normal or cancer) structure, both of which might hamper the models’ generalization capability. In addition, there is no outside validation of any of the models and this further limits their general applicability in clinical practice.

## 6. Conclusion

This research highlights the emerging value of transformer based models in medical image analysis, especially for critical applications such as lung cancer classification. While CNNs remain effective in capturing local visual features, our experiments confirmed that Vision Transformers (ViTs), when trained on tensor transformed input data, excel in capturing broader contextual patterns and subtle variations within CT and PET-CT scans. By evaluating a comprehensive suite of models under a consistent experimental framework including tensor-based input transformation as a fine-tuning step we established an evidence driven comparison between CNNs and ViT. These findings offer guidance for future development of AI-assisted diagnostic tools, emphasizing the trade offs between architectural complexity, generalization ability, and deployment feasibility.

Future work can explore hybrid models that combine the localized feature learning of CNNs with the global attention mechanisms of transformers. Additionally, incorporating clinical metadata, enabling multimodal learning, and expanding to 3D volumetric data or multi class classification may further enhance diagnostic precision. With continued innovation, transformer driven models augmented through fine tuned input processing hold potential for advancing personalized and accurate medical diagnostics, subject to further large-scale validation.
